# The Defective and Dependent


**Published:** 1912-03-02

**Authors:** 


### THE DEFECTIVE AND DEPENDENT.*

### VI.—The Education of the Deaf.

Formerly it was the custom to speak of those unfortunates who, by reason of deprivation of hearing were unable to speak, as "deaf and dumb"; but since the more general adoption of the oral system of teaching them language the latter designation, as also that of "deaf-mute," is falling into desuetude.

### Progress of Oral Instruction.

We have seen in a previous paper (November 18, 1911, p. 181) that the "German system" of oral instruction dates from the end of the eighteenth century; but it had few adherents in this country till 1871, when an Association for promoting the method was formed in London by the Baroness Meyer de Rothschild. At an International Congress on the Education of the Deaf, held at Milan in 1880, a resolution affirming the superiority of this method was agreed to, and in 1889 the Royal Commission on the Deaf, Blind, etc., went so far as to recommend that every deaf child should, unless physically or mentally disqualified, have full opportunity of being educated in the pure oral system. Transition from methods of signing and finger-spelling (the so-called "manual method") previously in vogue has, however, been but slow in this country, conservative opinion having tended to favour the retention in British institutions of a modicum of the old system in the form of what is called the "combined method." Experience shows, however, that except in the earliest stages of training such a combination is detrimental to progress, and the "pure oral" is now generally accepted as the only satisfactory system, except in a narrow margin of cases in which physical or mental defects preclude its application.

As in other forms of instruction, much depends on judicious classification of the pupils. According to Dr. J. Kerr Love, an experienced writer on the subject, complete deafness is comparatively rare. The classification of pupils which he recommends is as follows:—

(1) The *semi-mute*, *i.e.* children who have lost their hearing when from three to ten years old, and though quite deaf have a certain amount of speech left. These form from 5 to 10 per cent. of the children in a deaf-school.

(2) The *semi-deaf*, *i.e.* children possessing a considerable amount of residual hearing, the auditory organs having been to some degree damaged by disease. They retain, however, such an amount of residual hearing as to be able to distinguish certain sounds when spoken close to the ear. Such cases comprehend about 15 to 20 per cent. of the aggregate.

(3) The *average deaf-mute*. Such have no hearing for voice which can be turned to account, and form about 60 per cent. of those under instruction.

(4) The *mentally defective deaf-mute*, who suffer from ill-developed or damaged brain as well as from auditory defects, and require instruction by signs rather than on the oral method. These form from 10 to 20 per cent. of those presented for admission to deaf-schools.

It is computed that fully half of the cases requiring instruction are of congenital origin, or dating from so early an age as to give no time for speech to develop. Of acquired cases the most frequent causes in childhood are destructive inflammations following scarlet-fever, diphtheria, measles, influenza, and meningitis. Adenoid growths in children are a frequent cause of minor degrees of deafness, due to occlusion of the Eustachian tubes, and in such cases the removal of such growths is essential.

Deaf children are apt to be shallow breathers, by reason of the vocal apparatus not having been called into play, and deep breathing exercises are helpful as preliminary to speech training. In all cases the faculties of observation and perception should be cultivated from as early an age as possible.

The Act of 1893 provides for compulsory school attendance of deaf children from seven to sixteen years of age, but systematic training should begin earlier. The first steps in oral instruction are imitative exercises of simple gymnastic movements, and a special drill applicable to the tongue, lips, jaws, and larynx. Thus, following the teachers' example, the child is taught to extend its tongue so as to touch respectively the upper and lower lip, to close its teeth on the tip of the tongue with lips apart, to purse up its lips and puff, so as to prepare for the sound of P, etc. For the vowel sounds, the child is shown by the teacher the proper adjustment of the mouth and larynx and finally the latter emits the desired sound, having placed the pupil's hand on his throat so as to feel the accompanying vibration. The child, thus guided, tries to make the sound, and errors of tongue adjustment are rectified by the teacher with the aid of a spatula. Thus by persevering practice the elements of articulation are acquired, and by degrees the pupil learns to read off sounds, and afterwards words, from the teacher's lips.

### The Cost of Education.

The course of education is necessarily expensive, averaging about £23 per head per annum in the London day-schools; but in cases demanding so much individual attention one teacher cannot instruct more than eight or nine in a class. The power of communicating with his hearing fellows in ordinary language is, however, of inestimable benefit to the deaf pupil educated on the oral system, enabling him to obtain employment which would otherwise be closed to him, and so rescuing him from a position of dependence.

* Previous articles of this series have appeared in The Hospital of Nov. 4, Nov. 18, Dec. 2, Jan. 6, and Feb. 10.

